# Supplementary figures and images for: Quantifying the Dynamics of Coupled Networks of Switches and Oscillators
Source: PLoS One. 2012 Jan 5;7(1):e29497. doi: 10.1371/journal.pone.0029497 (PMC3252330; doi:10.1371/journal.pone.0029497)

$\kappa_{XX} = 0.01$

All Off

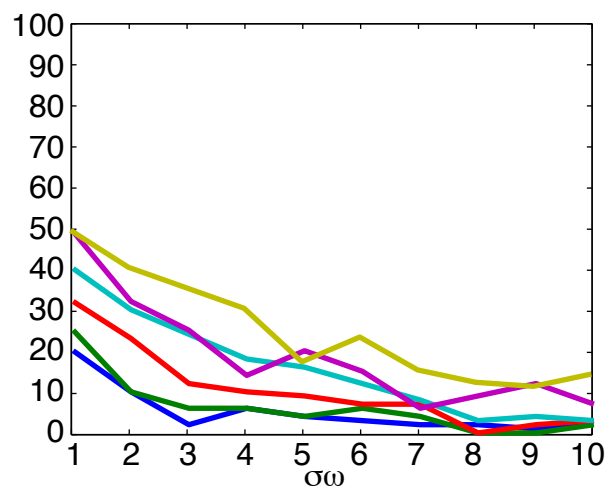

All On

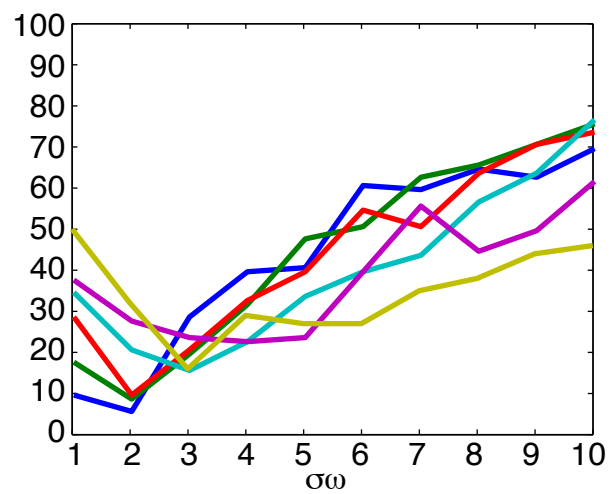

All Oscillating

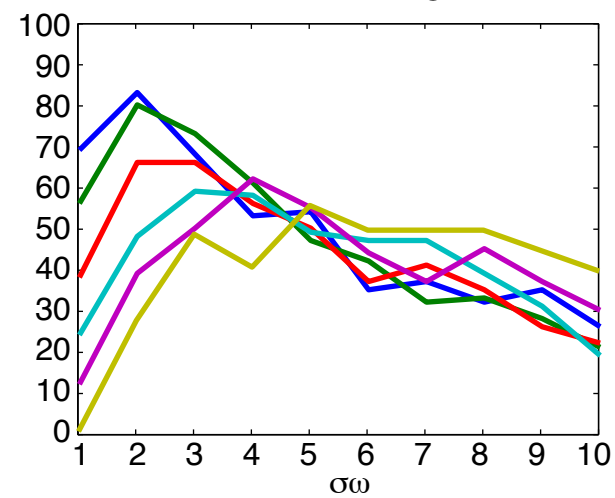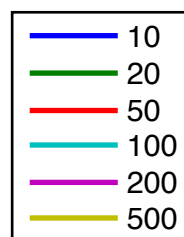

Supplement: Figure S1 — Dependence of dynamics on network size for . Number of simulations (of 100) for which the switches are off and oscillators are frozen (left panel), the switches are on and the oscillators are synchronized (center panel), and both the oscillators and switches have synchronized oscillations (right). (PDF) [file pone.0029497.s005.pdf]

$$\kappa_{XX} = 0.1$$

All Off

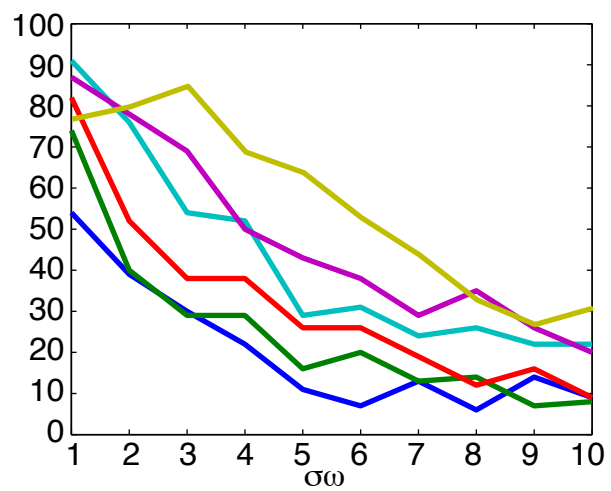

All On

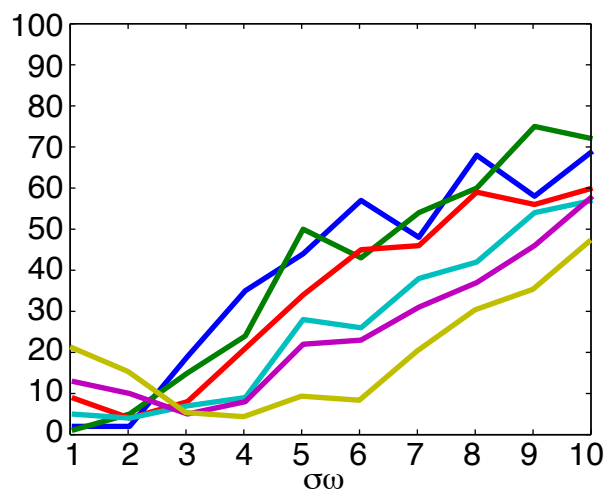

All Oscillating

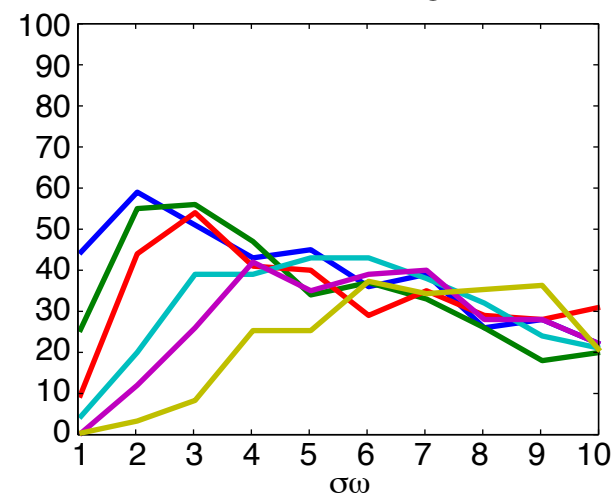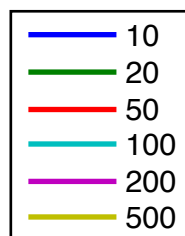

Supplement: Figure S2 — Dependence of dynamics on network size for . As for Figure S1. (PDF) [file pone.0029497.s006.pdf]

$$\kappa_{XX} = 1$$

All Off

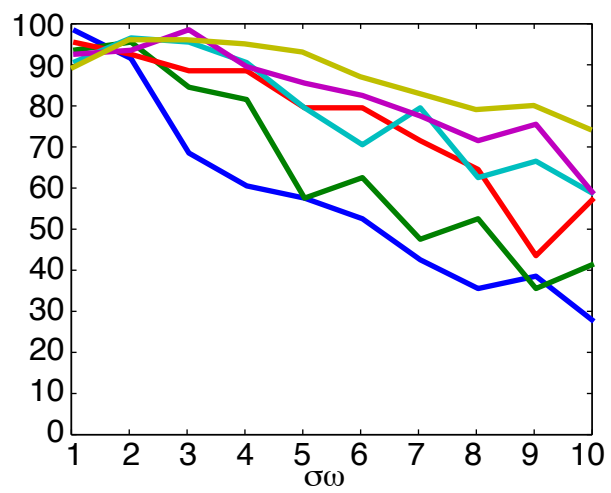

All On

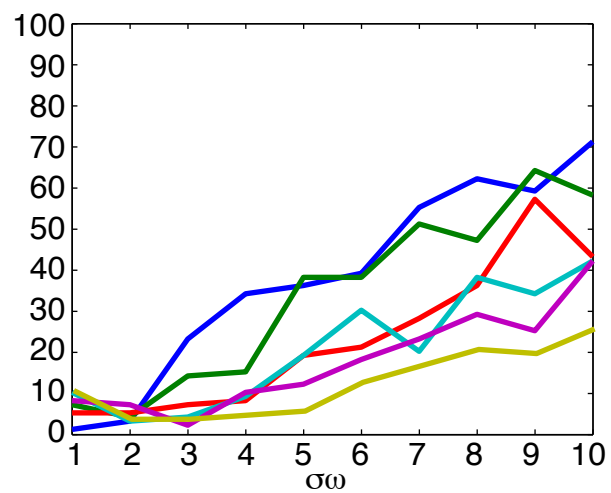

All Oscillating

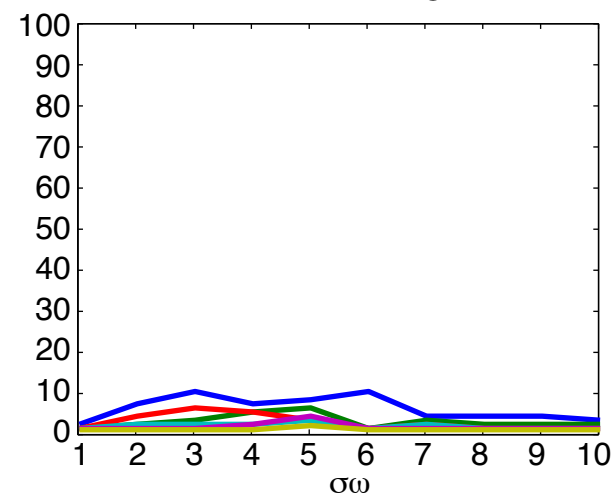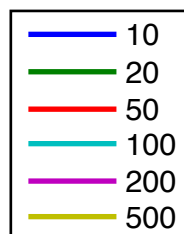

Supplement: Figure S3 — Dependence of dynamics on network size for . As for Figure S1. (PDF) [file pone.0029497.s007.pdf]
